# Long-term survival without graft-versus-host-disease following infusion of allogeneic myeloma-specific Vβ T cell families

**DOI:** 10.1186/s40425-019-0776-9

**Published:** 2019-11-14

**Authors:** S. Yado, G. Luboshits, O. Hazan, R. Or, M. A. Firer

**Affiliations:** 1 Chemical Engineering and Biotechnology, and Adelson School of Medicine, Ariel University, 40700 Ariel, Israel; 2 Ariel Center for Applied Cancer Research, Ariel University, 40700 Ariel, Israel; 3 Department of Bone Marrow Transplantation and Cancer Immunotherapy, Hadassah-Hebrew University Medical Center, Jerusalem, Israel; 4 Adelson Medical School, Ariel University, 40700 Ariel, Israel

**Keywords:** Bone marrow transplantation, Graft-versus-host disease, Graft-versus-myeloma, Adoptive allogeneic T-cell therapy; T cell–receptor Vβ families;

## Abstract

**Background:**

Despite chemo-induction therapy and autologous stem cell transplantation (ASCT), the vast majority of patients with Multiple Myeloma (MM) relapse within 7 years and the disease remains incurable. Adoptive Allogeneic T-cell therapy (ATCT) might be curative for MM, however current ATCT protocols often lead to graft versus host disease (GvHD). Transplanting only tumor reactive donor T cells that mediate a graft-versus-myeloma (GvM) but not GvHD may overcome this problem.

**Methods:**

We used an MHC-matched/miHA-disparate B10.D2 → Balb/c bone marrow transplantation (BMT) murine model and MOPC315.BM MM cells to develop an ATCT protocol consisting of total body irradiation, autologous-BMT and infusion of selective, myeloma-reactive lymphocytes of T cell receptor (TCR) Vβ 2, 3 and 8.3 families (MM-auto BMT ATCT).

**Results:**

Pre-stimulation ex vivo of allogeneic T cells by exposure to MOPC315.BM MM cells in the presence of IL-2, anti-CD3 and anti-CD28 resulted in expansion of the myeloma-reactive T cell TCRVβ 2, 3 and 8.3 subfamilies. Their isolation and infusion into MM-bearing mice resulted in a vigorous GvM response without induction GvHD and long-term survival. Repeated infusion of naïve myeloma-reactive T cell TCRVβ 2, 3 and 8.3 subfamilies was also effective.

**Conclusions:**

These data demonstrate that a transplantation protocol involving only selective tumor-reactive donor T cell families is an effective immunotherapy and results in long-term survival in a mouse model of human MM. The results highlight the need to develop similar ATCT strategies for MM patients that result in enhanced survival without symptoms of GvHD.

## Background

Survival of patients with multiple myeloma (MM) beyond 7 years remains rare even after autologous stem cell transplantation (ASCT) and treatment with novel agents [1]. Consequently, immunotherapies aimed at augmenting the anti-MM immune response, such as Adoptive Allogeneic T-cell Therapy (ATCT) have become attractive alternatives [2–4]. Much of the curative potential of allografts is attributed to the graft-versus-tumor (GvT) response that aims to destroy residual tumor cells that persist after induction therapy and ASCT [5]. Nonetheless, ATCT remains controversial [6] because the bulk donor T cells that mediate the GvT effect [7] can also induce graft versus-host disease (GvHD), a major cause of morbidity and mortality in ATCT recipients [8]. Various approaches to diminish the GvH response have had limited success [9–13].

Since GvT responses involve T-cell recognition of tumor-specific peptides presented by MHC molecules [14], it may be possible to identify and select donor T cells that provide beneficial GvT responses but minimal GvHD risk. In this regard, immune-transcriptome analyses of T cell receptor (TCR) Vβ CDR3-size and sequence is being used to characterize alloreactive versus tumor-specific T-cell responses. Korngold and colleagues identified donor alloreactive CD8+ and CD4+ Vβ families responsible for GvHD in several animal models of bone marrow transplantation (BMT) [15–18]. Binsfeld et al. studied the Vβ families involved in the GvM and the GvH response in an MM-BMT model, finding the Vβ 2, 3 and 8.3 families of T cells as those specifically involved in the GvM response [19]. The implication of these results would be that myeloma-specific T cell subfamilies might be positively selected from the donor inoculum and infused to myeloma patients post ASCT, to afford separation of allo- from tumor-reactive T cells without the prior need to define specific target antigens.

To test this rationale, we used the allogeneic B10.D2 → Balb/c BMT model with MOPC315.BM myeloma cells. We first demonstrated that myeloma bearing-Balb/c mice initially respond clinically to irradiation and auto-BMT but eventually relapse, similar to MM patients undergoing induction therapy and ASCT. By then infusing the animals with B10.D2 T cells from only the TCR Vβ 2, 3 and 8.3 families appropriately pre-activated in vitro, we saw a vigorous GvM response without any clinical or histological signs of GvHD or disease relapse, which translated into long-term, disease-free survival. These data highlight the possibility that tumor-specific ATCT may lead to long-term disease-free survival without GvHD in patients with MM.

## Methods

### Ethical statement

All experimental procedures were performed in accordance with protocols approved by the Ariel University Institutional Animal Care and Use Committee. Animal welfare was assessed at least daily. After completion of experiments mice were euthanized in a CO_2_ chamber.

### Animals

Balb/c (H-2^d^) mice were obtained from Envigo Laboratories (Jerusalem, Israel). B10.D2 (H-2^d^) mice were purchased from Jackson Laboratories (Bar Harbor, ME, USA) and bred in the Ariel University Animal Facility. For all experiments, male mice between the ages of 10 and 14 weeks were used as donors and recipients. Treated mice were kept in a pathogen-free environment in autoclaved microisolator cages and were provided with acidified (pH 2.5) water and autoclaved food ad libitum.

### Myeloma cell line and model

MOPC315.BM cells [20] was kindly provided by Prof. Bjarne Bogen (University of Oslo, Norway). They were cultured at 37 °C in 5% CO_2_ in RPMI 1640 (Sigma-Aldrich, Rehovot, Israel) supplemented with 10% FBS, 1% MEM NEAA 100x (Gibco), 0.005% 1 M I-thioglycerol, 0.03% Gensumycin 40 mg/ml (Sigma-Aldrich) and 2 mM L-glutamine (Biological Industries, Beit Haemek, Israel). I.v. injection of MOPC315.BM cells results in tumor development in the bone marrow (BM) and spleen and is associated with osteolytic lesions, validating the model as resembling human MM disease [21]. In advanced disease stages (within 3–4 weeks), the mice develop paraplegia through spinal cord compression. They were sacrificed when presenting signs of paraplegia, deterioration of general condition or apathy.

### Experimental transplantation design (Fig. 1a)

Balb/c mice were injected i.v. into the tail vein with 1 × 10^6^ MOPC315.BM cells in 100 μl RPMI 1640. Preliminary experiments showed that paraplegia developed 38 days post injection (Additional file 1: Figure S1). At day 35, mice were irradiated with 6.5 Gy (Total Body Irradiation) using an X ray source (Kimtron Polaris 320) and injected 6 h later with an infusion of syngeneic 10 × 10^6^ BM and 70 × 10^6^ spleen cells from healthy Balb/c donors (Day 0). BM cells were collected by flushing the femurs and tibias into sterile PBS. Spleens were crushed through a 70-μm cell strainer into sterile PBS (Biological Industries) and Red blood cells lysed (RBC lysis buffer, eBioscience, San Diego, USA). Animals that received this transplant protocol are referred to as “MM-Auto-BMT” mice. For ATCT experiments, on day 10 and in some experiments also on day 17 post MM Auto-BMT, mice received an infusion of 1 × 10^6^ or 2.5 × 10^6^ B10.D2 or Balb/c Vβ 2, 3 and 8.3 positive T cells (MM-Auto-BMT-ATCT group) or unselected spleenocytes. These Myeloma-reactive T cells (MT-cells) were isolated with antibody-coated magnetic beads from donor spleenocytes, either pre-activated by MOPC315.BM cells or not (naïve cells) (see below).

**Fig. 1:**
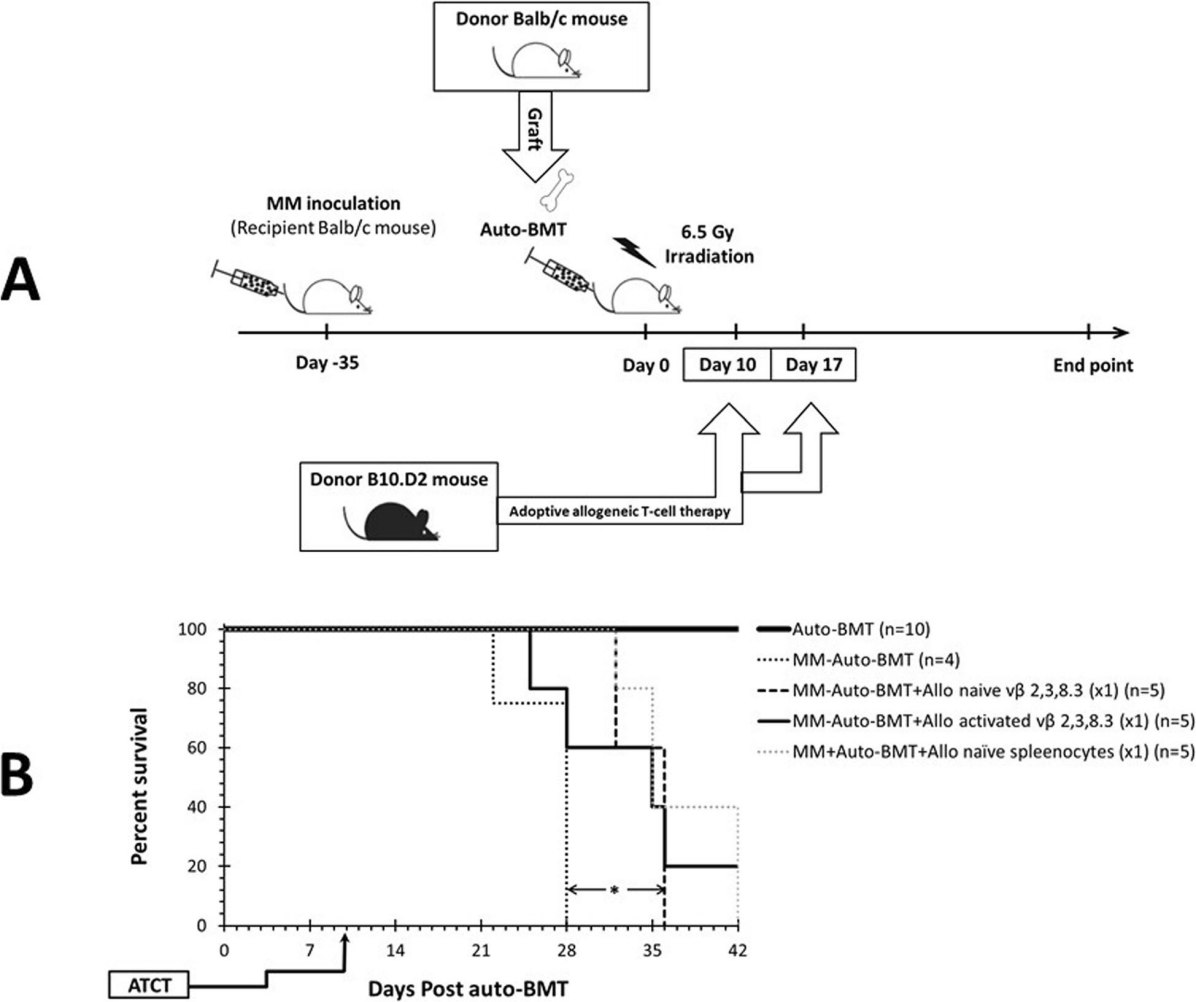
**a** Experimental design and monitoring of a mouse model of multiple myeloma to test Graft versus Myeloma and Graft versus Host Disease effects following allogeneic adoptive T cell therapy. MM-bearing Balb/c (Additional file 1: Figure S1) were irradiated and transplanted by i.v. injection of both BM cells and spleenocytes from healthy Balb/c donor mice. Immune reconstitution was validated by evaluation of CD4+ and CD8+ lymphocyte population representation in BM and spleen (Additional file 1: Figure S4). These animals were referred to as “MM-Auto-BMT” mice. For ATCT experiments, on day 10 and in some experiments also on day 17 post Auto-BMT, mice received an infusion of B10.D2 or Balb/c Vβ 2, 3 and 8.3 positive T cells (MM-Auto-BMT-ATCT group). These Myeloma-reactive T cells (MT-cells) were isolated with antibody-coated magnetic beads from donor spleenocytes, pre-activated by MOPC315.BM cells or not (target-naïve cells) (see text). **b** Survival of myeloma bearing Balb/c mice treated by irradiation and autologous bone marrow transplant (Auto-BMT) and then allogeneic lymphocyte infusion. The presented results represent the average of two independent experiments. On day 10 after Auto-BMT, mice were injected i.v. with naïve or MOPC315.BM (target cell) activated B10.D2 Vβ 2, 3 and 8.3 T cells. Recipient mice were sacrificed when severe GvHD symptoms (GvHD score > 8/10), myeloma symptoms (e.g. paraplegia) or apathy were present. Statistical significance between survival curves was determined using the Log-Rank test. MM-Auto-BMT versus MM-Auto-BMT + Allo naive vβ 2, 3, 8.3 (× 1), *p = 0.005; MM-Auto-BMT versus MM-Auto-BMT + Allo activated vβ 2, 3, 8.3 (× 1), *p* = 0.137; MM-Auto-BMT + Allo naive vβ 2, 3, 8.3 (× 1) versus MM-Auto-BMT + Allo activated vβ 2, 3, 8.3 (× 1), *p* = 0.862

Recipient mice were checked daily for morbidity and mortality and sacrificed after appearance of symptoms of myeloma (See Additional file 1: Video S1) and/or GvHD. Three mice from each experimental condition were euthanized on days − 2 (before), + 7 and + 14 post auto-BMT and at the end point. BM and spleens were harvested and analyzed by flow cytometry for the presence of MOPC315.BM cells and to monitor repopulation of T cell subsets. Before sacrifice, a blood sample was obtained for measurement of M315 myeloma paraprotein.

### In vitro T-cell activation and cytotoxicity

Target MOPC315.BM cells were treated for 2 h with 5 μg/ml of mitomycin C (Sigma-Aldrich) to arrest cell growth. Following washing, they were then co-cultured in complete medium (RPMI 1640, 10% FBS, 1% Penicillin/Streptomycin, 2 mM L-glutamine and 50 μg/mL 2-mercaptoethanol) supplemented with recombinant IL--2 (20 U/mL, Biolegend) for 4 days at a ratio of 20:1 with 5 × 10^6^ spleenocytes isolated from healthy B10.D2 or Balb/c mice. In later experiments, cells were co-cultured for 2 days in medium containing 50 U/mL rIL-2, anti-CD3 (5 μg/ml) and anti-CD28 (2 μg/ml) (eBioscience) antibodies. Vβ 2, 3 and 8.3 T cells activated with this second protocol are referred to as “IL-2/Ab” activated allo- (B10.D2) or auto- (Balb/c) MT-cells. Following co-culture, spleenocytes were analyzed by flow cytometry and used for cytotoxicity assays. MT cells were isolated by incubation with 0.5 mg/ml of PE-conjugated monoclonal antibodies: anti-Vβ 2 (clone B20.6), anti-Vβ 3 (clone KJ25), and anti-Vβ 8.3 (clone 1B3.3) (BD Pharmingen, San Jose, CA) followed by anti-PE mAb–conjugated magnetic beads and separation using the SuperMacs system (Miltenyi Biotec, Auburn, CA). The positive fraction was typically > 90% PE positive as determined by flow cytometry.

To test the cytotoxicity of donor B10.D2 or Balb/c MT cells, 10^7^ fresh MOPC315.BM target cells/mL were labeled with 1 μM carboxyfluorescein succinimidyl ester (CFSE) (eBioscience) for 10 min at RT. The reaction was stopped by addition of 4–5 volumes of cold complete media and 5-min incubation on ice. After washing with complete medium, the target cells were resuspended in complete medium at 1 × 10^6^ cells/mL dispensed into 96-well microtiter plates (100 μL/well). MT cell populations were added in 20:1 10:1 and 5:1 effector-to-target ratios in a total volume of 250 μL complete medium and plates were incubated at 37 °C in 5% CO_2_ for 4 h. The percentage of MOPC315.BM cell death was evaluated by staining with Sytox blue (1 μM, Molecular Probes) and flow cytometry. Target cells incubated without effector cells (to measure spontaneous death) were used as control.

### GvHD clinical-scoring system

GvHD symptoms were evaluated with a scoring system adapted from Cooke et al. [22]. The score is based on weight loss (< 10% = 0; 10–20% = 1; > 20% = 2), hunched back posture (normal = 0; hunched-back while resting = 1; persistent = 2), general activity (normal = 0, reduced activity = 1, apathy = 2), alopecia (normal = 0, < 1 cm^2^ = 1, > 1 cm^2^ = 2) and skin fibrosis (normal = 0, fibrosis = 1; scabs = 2) with a maximum score of 10. Each animal’s condition was monitored daily, and the GvHD score was calculated at least 3 times per week. Mice were sacrificed if they reached a score of 8/10 or when apathetic.

### Flow cytometry

Fc receptor binding was blocked by incubation with anti-CD16/CD32 antibodies (clone 93, eBioscience) for 5 min at RT. The cells were then incubated for 30 min at 4 °C with specific antibodies (anti-CD3e/APC (145-2C11), anti-CD4/FITC (GK1.5), anti-CD8/eFluor506 (53–6.7), anti-CD25/PE-Cy7 (PC61.5), (eBioscience); anti-CD3/PE (17A2); anti-CD69/Pacific blue (H1.2F3); anti-B220/ PE-Cy7 (RA3-6B2) (Biolegend (San Diego, CA); anti-IgA/FITC (C10–3) (BD Biosciences) and CD138/APC (REA104) (Miltenyi Biotec) in PBS/ 3% FBS, washed and resuspended in cold PBS. The data were acquired by a CytoFLEX (Beckman Coulter) flow cytometer and analyzed using FlowJo software.

### Histology

Approximately 2 cm^2^ of shaved skin from the interscapular region (GvHD–target organ) and representative spleen and colon samples were collected from sacrificed mice, fixed in 10% formalin, paraffin embedded, cut into 5-μm-thick sections and stained with hematoxylin and eosin. Histological processing and assessment was performed by Patho-Lab Diagnostics (Nes Ziona, Science Park, Israel).

### Serum paraprotein quantitation

Paraprotein production by MOPC315.BM cells was evaluated by ELISA [23]. Briefly, 96 well Nunclon ELISA plates were coated with 2 μg/ml of anti-MOPC315.BM paraprotein idiotype (Ab2.1–4) (kindly provided by Prof Bjarne Bogen, University of Oslo, Norway) at 4 °C overnight. Wells were blocked with PBS/0.02% sodium azide/1% BSA, washed and incubated for 2 h at 37 °C with serum samples or standard paraprotein (ranging from 400 to 0.39 ng/ml) diluted in PBS/ 0.02% sodium azide/0.1% BSA/0.1% Tween 20. Then, the plates were incubated with 1 μg/ml biotinylated rat anti-mouse IgA (clone C10–1, BD Pharmingen, Germany) for 1 h at RT, washed, incubated with streptavidin- HRP (1:2000; Sigma-Aldrich) for 1 h at RT and washed again. TMB substrate (Merck Millipore, Billerica, MA, USA) was added for 10-min, the reaction was terminated with H_2_O_2_ and absorbance measured at 450 nm with a TECAN Infinite M200 ELISA reader.

### Statistics

The Log-Rank test was used to compare the Kaplan-Meyer survival plots. Median survival times (MST) were calculated, and a *p* value ≤0.05 was considered statistically significant. Statistical significance between groups was determined using a Student *t* test. A *p* value ≤0.05 was considered statistically significant.

## Results

### B10.D2 Vβ 2, 3 and 8.3 T cells families induce GvM but not GvHD

On day 10 after Auto-BMT, but prior to the time of their expected relapse, MM-Auto-BMT mice received a T cell infusion comprising donor B10.D2, or Balb/c MT cells (Allo-MT cells or Auto-MT cells respectively) or unselected spleenocytes.

MM-Auto-BMT control mice who received sham (no lymphocyte) infusion succumbed to MM with an MST of 28 d, while those who received Allo-MT experienced significantly extended survival (MST = 28 d versus MST = 36 d, respectively; *p = 0.005) and did not develop signs of GvHD. However, 100% of these mice eventually succumbed to myeloma progression (Fig. 1b). MM-Auto-BMT mice who received unselected B10.D2 spleenocytes also experienced extended survival. However, they developed typical signs of chronic GvHD and succumbed to the disease with a MST of 35 d.

We tested whether ex vivo activation of Allo-MT cells prior to injection could boost the GvM response with minimal GvHD complications. B10.D2 spleen cells were co-cultured with Mitomycin C-pretreated myeloma cells at a ratio of 20:1 in medium supplemented with 20 U rIL-2. Flow cytometry showed an expansion of both CD8^+^ and CD4^+^ T cell populations and a significant increase in activated CD4^+^ and CD8^+^vβ (2, 3, 8.3)^+^ T cells, confirming their reactivity against myeloma target cells (Additional file 1: Figure S2). Therefore, 1 × 10^6^ Allo-MT cells, either naïve or MOPC315.BM-activated, were injected into MM-Auto-BMT mice on day 10 after the autograft. This treatment also extended the MST to 35d and there were no signs of GvHD but again, 80% of the mice eventually succumbed to myeloma progression. There was no significant difference in MST between mice that received naïve or MOPC315.BM-activated Allo-MT cells (MST = 35 d versus MST = 36 d, respectively; *p* = 0.862) (Fig. 1b).

**Fig. 2:**
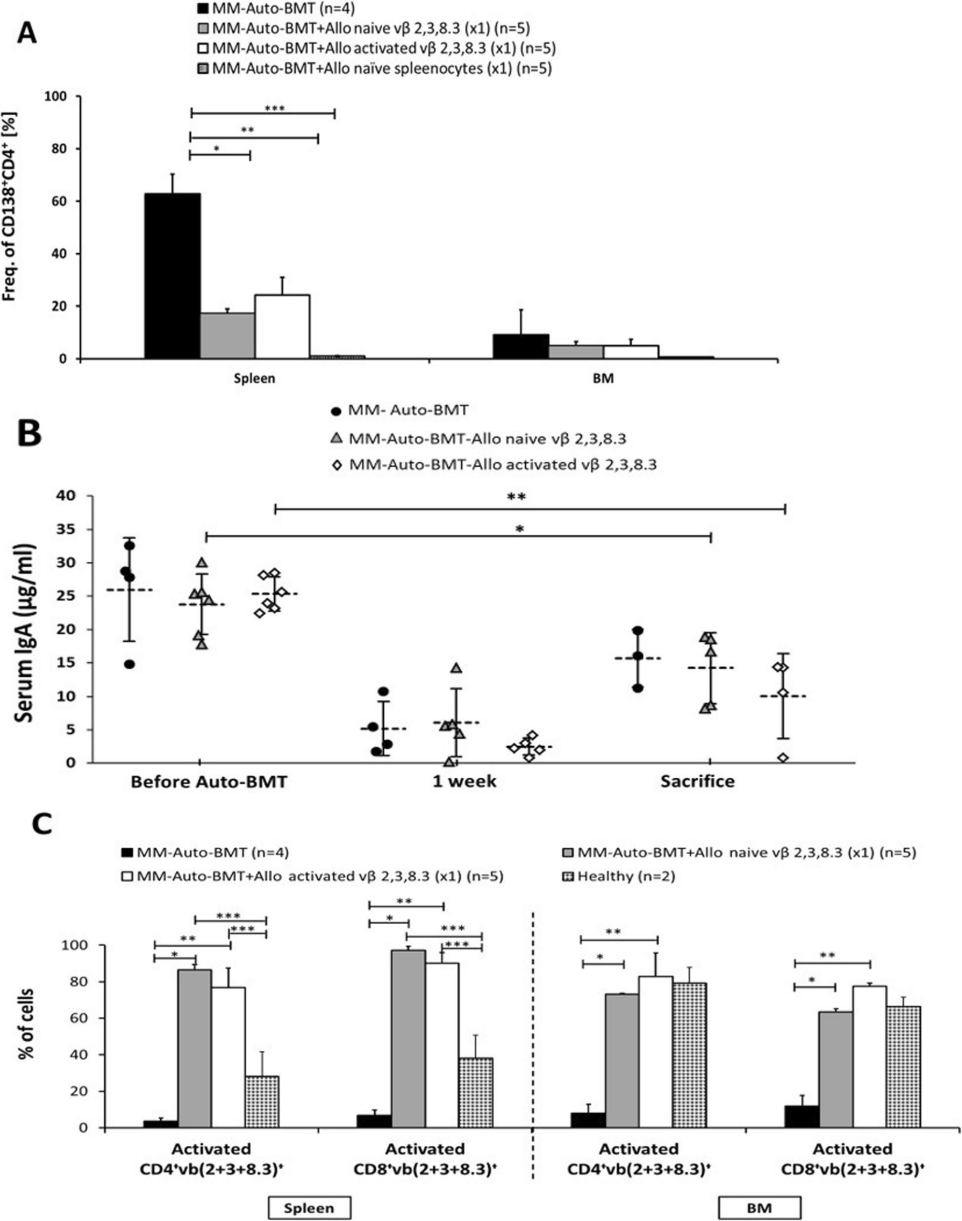
Involvement of vβ TCR CDR3 2, 3, 8.3 T cell families in the graft-versus-myeloma effect in myeloma bearing Balb/c mice treated by irradiation, Auto-BMT and then allogeneic lymphocyte infusion. Recipient mice were sacrificed when severe GvHD symptoms, myeloma symptoms or apathy were present. Flow cytometry staining was performed on cells from spleen and bone marrow at sacrifice. **a** Infiltration of MOPC MM cells in the bone marrow and spleen identified as CD138^+^CD4^+^ double positive cells. *p = 0.0006. **p = 0.0018 (Student *t* test). **b** Paraprotein serum IgA quantitation (μg/ml) by ELISA before Auto-BMT, 1 week after and at sacrifice. *p = 0.0003. **p = 0.005 (Student *t* test). **c** vβ(2 + 3 + 8.3)^+^ T cell populations in the graft-versus-myeloma effect. Shown are percentages of activated CD4^+^vβ(2 + 3 + 8.3)^+^ T cells (CD69+ within CD4^+^vβ(2 + 3 + 8.3)^+^ T cells) and activated CD8^+^vβ(2 + 3 + 8.3)^+^ T cells (CD69+ within CD8^+^vβ(2 + 3 + 8.3)^+^ T cells) in the spleen (left panel) BM (right panel) in the MM-Auto-BMT, MM-Auto-BMT + Allo naive vβ 2, 3, 8.3 (× 1) group, MM-Auto-BMT + Allo activated vβ 2, 3, 8.3 (× 1) or in healthy Balb/c mice. *p < 0.0001; **p < 0.0001; ***p < 0.05 (Student *t* test)

At sacrifice, all ATCT treated groups who received either naïve or activated Vbeta T cells or naïve unselected spleenocytes, had significantly lower myeloma cell infiltration in the spleen compared to the control group (Fig. 2a, *p = 0.0006, **p = 0.0018, ***p = 0.0001 respectively) and accordingly they produced less serum paraprotein (Fig. 2b). Percentages of activated CD4^+^ and CD8^+^ T cells were significantly higher in the BM and spleen of mice that received MT cells (Fig. 2c), suggesting that these cells might be responsible for the observed GvM effect. These data indicate that infusion of donor myeloma-reactive T cells can provoke a potent GvM effect, without GvHD, leading to extended, but nonetheless limited, overall survival.

### Improved activation of B10.D2 Vβ 2, 3 and 8.3 T cells

**Fig. 3:**
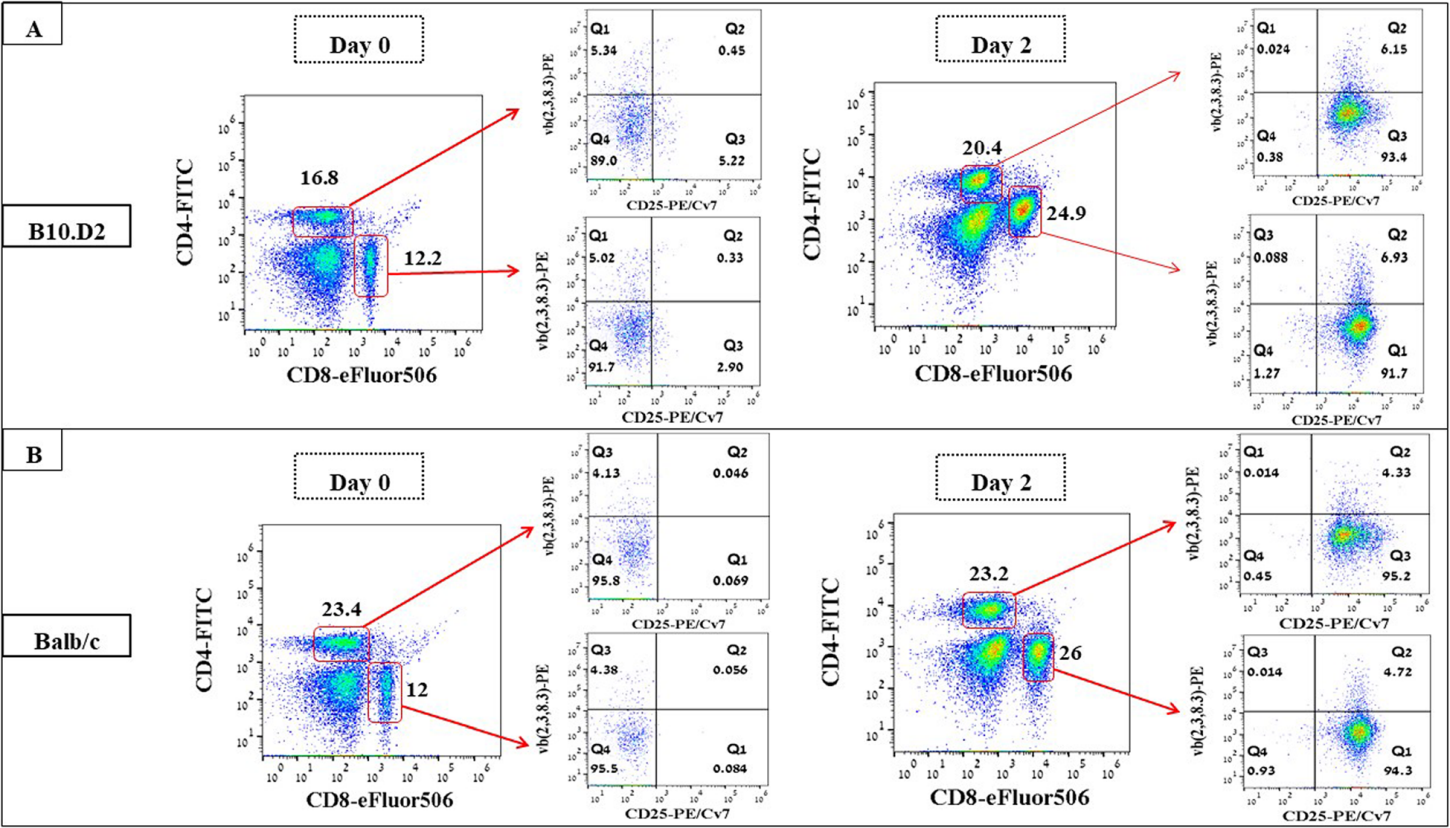
Flow cytometric T cell phenotyping before (day 0) and after in vitro activation (day 2) of B10.D2 (**a**) and Balb/c (**b**) Spleenocytes with Mitomycin-C-treated MOPC315.BM cells in medium containing 50 U/mL rIL and CD3/CD28 antibodies. The gating strategy is shown by the red arrows. The resulting CD4^+^ and CD8^+^ populations were further gated based on positivity for vβ (2, 3, 8.3) and CD25 (right panels). T cell activation was assessed by CD25 expression. One representative example of 2 independent experiments is shown

We questioned whether a more clinically effective GvM (no GvHD) response might be obtained by improving the ex vivo activation protocol of the Allo-MT cells. Therefore, spleenocytes from B10.D2 or Balb/c mice were stimulated by Mitomycin-C-treated MOPC315.BM cells for 2 days in medium containing 50 U/mL rIL-2 and anti-CD3/anti-CD28 antibodies (referred to as IL-2/Ab) [24]. This protocol resulted in an expansion of CD4^+^ T cells and a significant expansion of CD8^+^ T cells (2-fold) in B10.D2 spleenocyte cultures (Fig. 3). In Balb/c spleenocyte cultures, only CD8^+^ T cells expanded. There was a strong activation induced CD25 expression on MT cell families in both B10.D2 and Balb/c spleenocyte cultures. The cytotoxic capacity of these activated lymphocytes was validated by co-culturing them in different ratios with CFSE-labeled fresh MOPC315.BM. The degree of target cell killing was depended on the effector:target cell ratio with the best specific lysis (24% for B10.D2 and 19% for Balb/c) achieved at the highest E/T ratio tested (20:1) (Additional file 1: Figure S3).

### Enhanced MT cell activation leads to long-term survival without GvHD

**Fig. 4:**
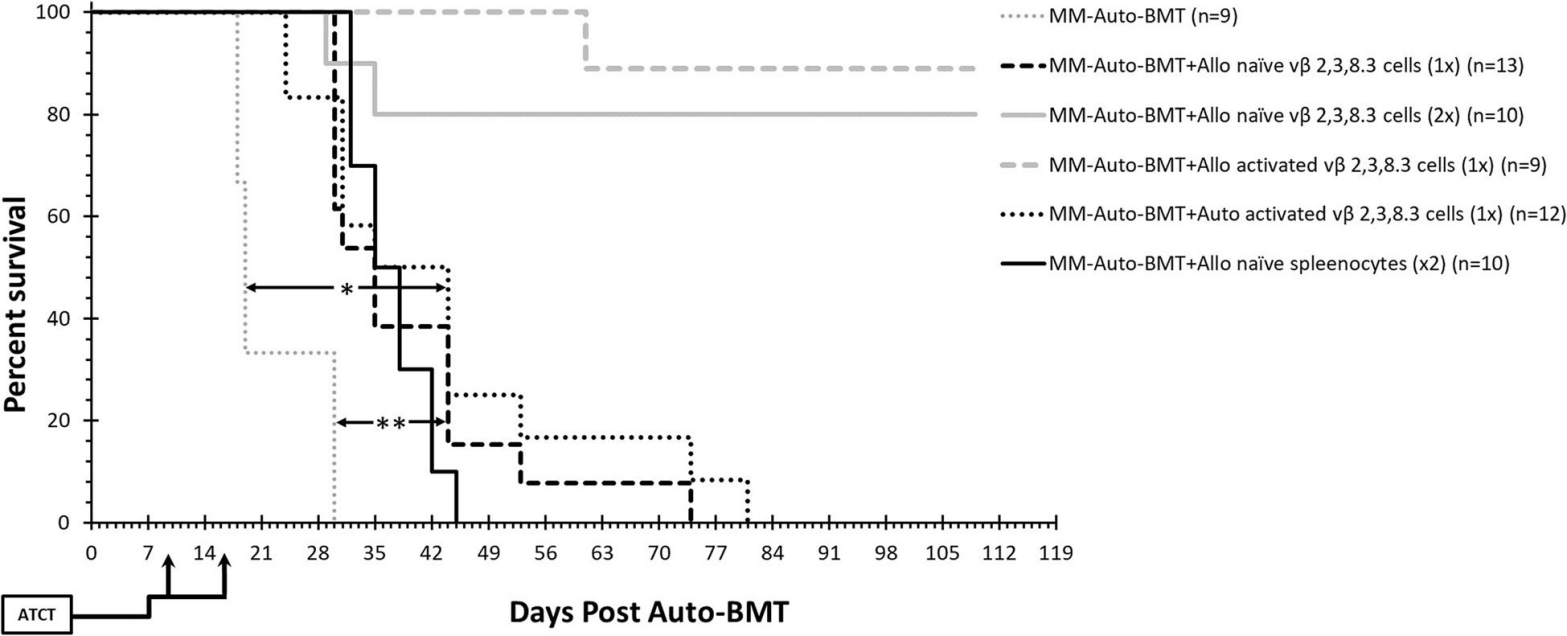
Survival curves of myeloma bearing Balb/c recipient mice treated by irradiation, Auto-BMT and then allogeneic or autologous lymphocyte infusion. The presented results represent the average of two independent experiments. On day 10 and/or day 17 after Auto-BMT, recipient mice were injected i.v. with naïve or activated B10.D2 / Balb/c Vβ 2, 3 and 8.3 T cells. Recipient mice were sacrificed when severe GvHD symptoms, myeloma symptoms or apathy were present. Statistical significance between survival curves was determined using the Log-Rank test. MM-Auto-BMT versus MM-Auto-BMT + Auto activated vβ 2, 3, 8.3 (× 1), *p < 0.0001; MM-Auto-BMT versus MM-Auto-BMT + Allo naive vβ 2, 3, 8.3 (× 1), **p = 0.0001

The effect of the IL-2/Ab activated MT cells was then tested in vivo*.* On day 10 after auto-BMT, MM-Auto-BMT mice received 2.5 × 10^6^ of IL-2/Ab activated Allo- or Auto-MT cells (The equivalent dose of these cells found in healthy B10.D2 and Balb/c mouse spleens as determined by flow cytometry). As shown in Fig. 4, 88% of mice who received IL-2/Ab activated Allo-MT cells survived at least 109 days post auto-BMT. Significantly, none of these animals developed symptoms of GvHD. Infusion of IL-2/Ab activated Auto-MT cells also provided a significant, albeit short-term GvM effect (MST = 44 d versus MST = 19 d, respectively; *p < 0.0001), although 100% of these mice eventually succumbed to myeloma progression.

We also tested whether an additional dose of naïve Allo-MT cells might circumvent the need for pre-activation. As shown in Fig. 4, mice who received an additional infusion of these cells on day 17 displayed no symptoms of GvHD and 80% of them had survived by the end of the experiment (109 days). Mice who received unselected B10.D2 spleenocytes displayed the typical signs of chronic GvHD and succumbed to the disease with a MST of 35 days.

**Fig. 5:**
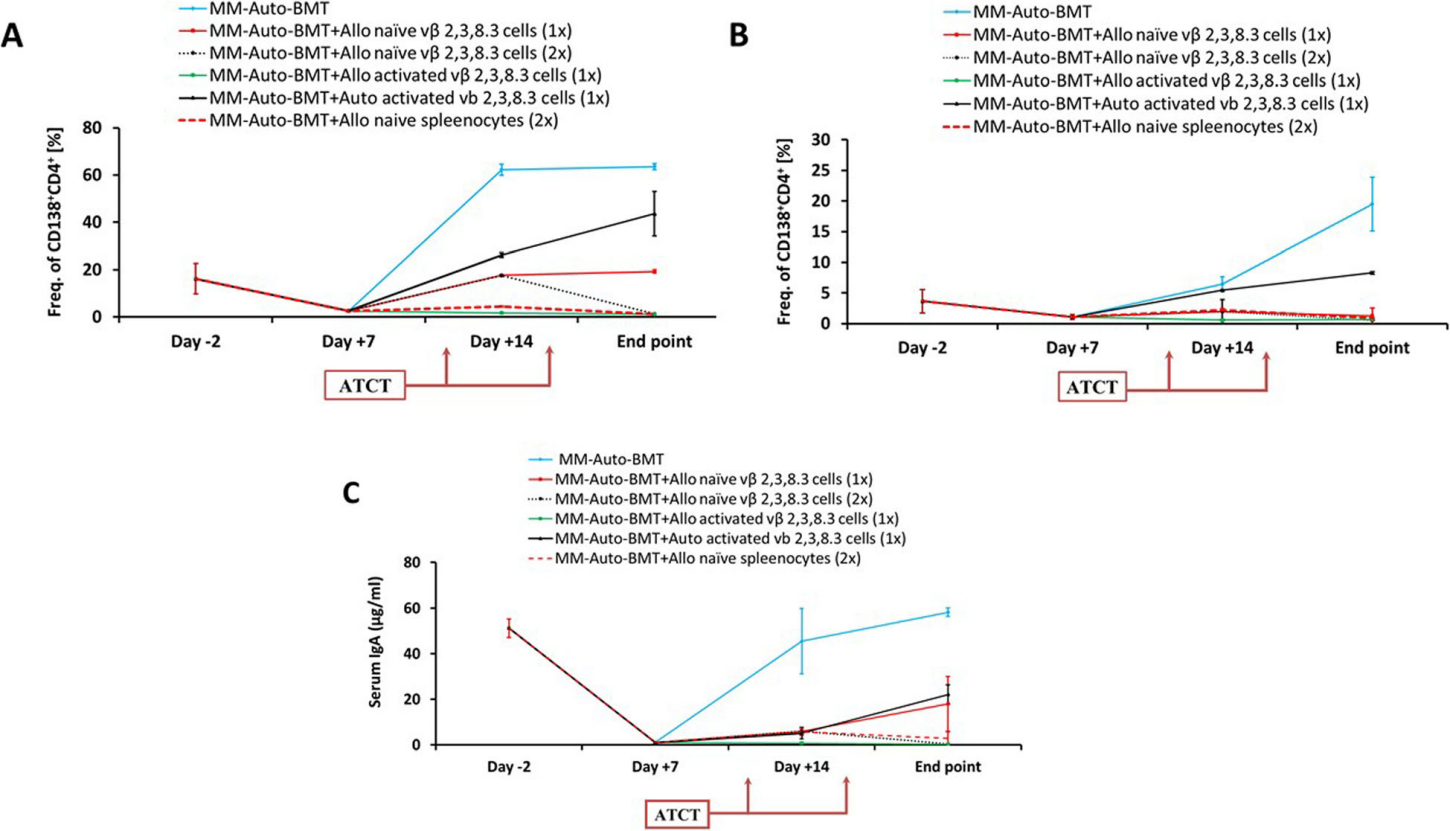
Correlation between MM disease parameters, GvHD and Adoptive T cell Therapy strategies**.** Infiltration of MM cells in spleen (**a**) and bone marrow (**b**) and levels of M315 myeloma protein (μg/ml) in sera of mice (**c**) for MM-Auto-BMT control group and ATCT groups. Three mice per group were sacrifice 2 days before transplantation, 10 and 17 days after transplantation and at the end point. Data are expressed as the mean ± SD. MOPC cells identified as CD138^+^CD4^+^ double positive cells by flow cytometry staining

**Fig. 6:**
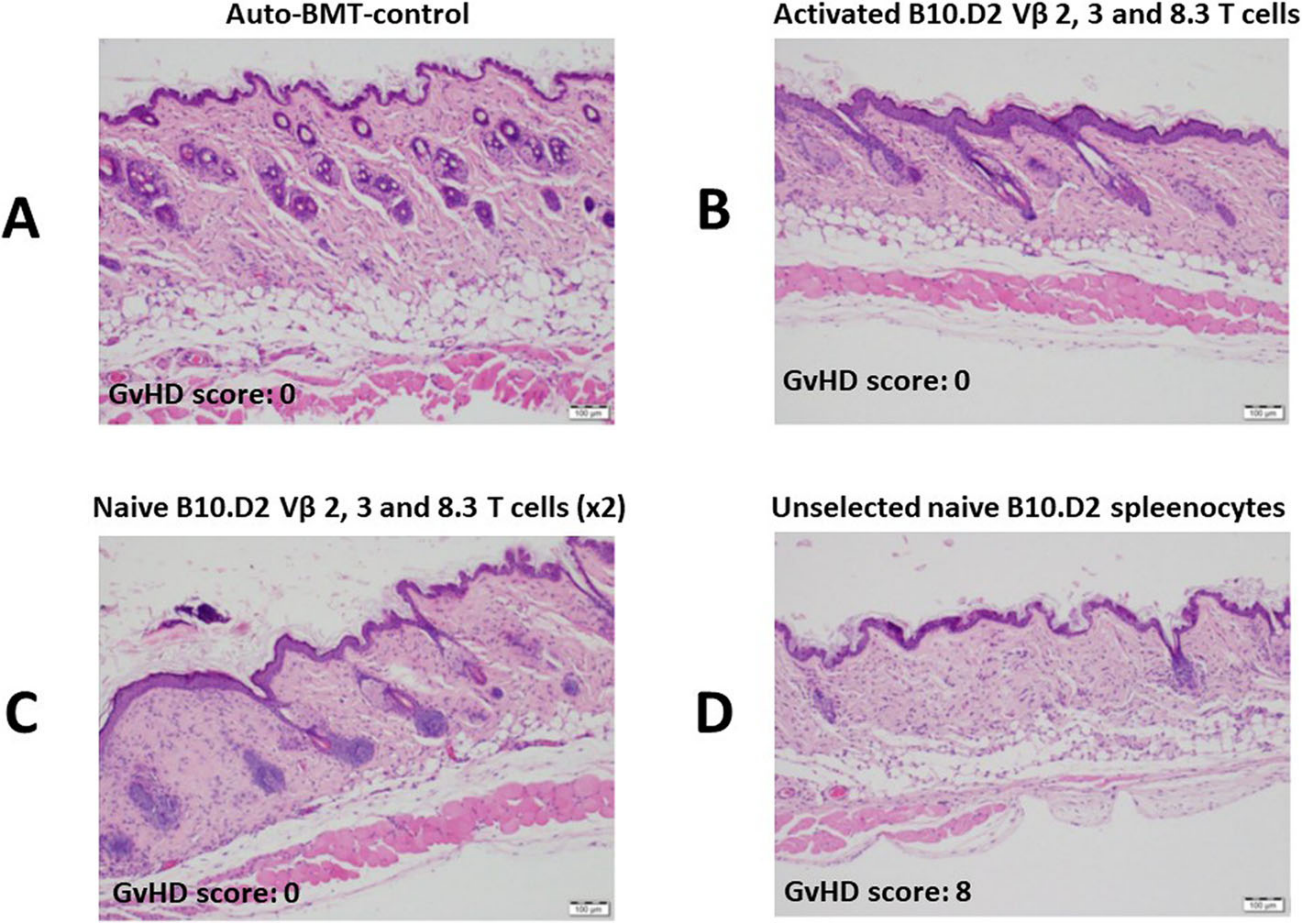
Histologic changes in skin. Comparive histology of skin tissue collected from the interscapular region of mice who received auto-BMT alone, IL-2/anti-CD3/anti-CD28 activated B10.D2 Vβ 2, 3 and 8.3 T cells, naive B10.D2 Vβ 2, 3 and 8.3 T cells (× 2) or unselected naive B10.D2 spleenocytes. H&E reveals normal epidermis in the samples of mice who received auto-BMT alone (**a**), IL-2/anti-CD3/anti-CD28 activated B10.D2 Vβ 2, 3 (**b**) and 8.3 T cells and naive B10.D2 Vβ 2, 3 and 8.3 T cells (× 2) (**c**), whereas there is decrease in follicular units, increased collagen density with increased cellularity (fibrosis) in the sample of mice who received unselected naive B10.D2 spleenocytes (**d**). Original magnification × 10. GvHD disease score (based on weight loss, hunched back posture, general activity, alopecia and skin fibrosis, on a scale of 0–10), was calculated

The effect of these different infusions on disease burden was tracked. On day − 2 before the auto-BMT, MM cells were detected in spleen and BM while at day + 7 after auto-BMT, there was a decrease in of MM cells (Fig. 5a–b), probably due to irradiation. At day + 14, the percentage of MM cells increased in all groups, except in those that received IL-2/Ab activated Allo-MT cells or naïve unselected B10.D2 spleenocytes on day 10 after auto-BMT. At the end point of each group, MM cell infiltration had further increased in the control mice and in those that received activated Auto- or naïve Allo-MT cells only on day 10. Conversely, MM cells were essentially undetectable in mice who received two infusions of naïve Allo-MT cells or unselected B10.D2 spleenocytes and in those that received IL-2/Ab activated Allo-MT cells only on day 10. These results were highly correlated with paraprotein serum M315 levels (Fig. 5c). Histo-pathological examination of interscapular skin tissues collected at experiment end points showed that Auto-BMT mice had no change in skin architecture (Fig. 6a) and were similar to normal mice (not shown). Likewise, mice who received either IL-2/Ab activated (Fig. 6b) or naive Allo-MT cells (× 2) (Fig. 6c) had a normal epidermis, whereas mice who received unselected B10.D2 spleenocytes exhibited classical chronic GvHD pathology (Fig. 6d). Liver and colon samples showed no histological signs of GvHD (data not shown). Taken together, these findings highlight that ATCT with appropriately pre-activated donor B10.D2 T cell families can produce a long-lasting GvM response in the complete absence of GvHD in MM-bearing Balb/c mice. Impressive results can be also be obtained with repeated infusion of naïve MM-specific donor B10.D2 T cell families.

## Discussion

Allogeneic immunotherapy remains the only potentially curable treatment for MM but the frequent co-development of GvHD after this type of therapy severely limits its clinical application. Unfortunately, the clinical success of strategies to reduce GvHD while retaining the GvT response have been limited [3, 10, 25].

Korngold and colleagues demonstrated that CDR3-size spectratyping of the TCRVβ-chain can characterize and differentiate alloreactive from GvT-specific T-cell repertoire responses, highlighting the potential for tailoring the donor inoculum to target only the recipient’s malignant cells [18, 26, 27]. Our aim was to apply TCRVβ-chain CDR3-sizing to allo-immunotherapy, by positively selecting MM-specific donor T cell families and testing if their infusion could affect a clinically relevant GvM response without inducing GvHD.

We used the well-established MHC-matched/ miHA-disparate B10.D2 → Balb/c BMT model [28] and induced MM in recipients by injecting MOPC315.BM MM cells [21]. MM-bearing mice were treated by total body irradiation and auto-BMT, followed by infusion of donor myeloma-reactive TCR Vβ^+^ T cells (Vβ 2, 3 and 8.3 families) identified previously [19]. In vitro experiments (Additional file 1: Figure S3) and the finding that the transplantation of these cells induced life-prolonging GvM effects but with no clinical (Fig. 4), biomarker (Fig. 5) or histological (Fig. 6) signs of GvHD indicates that these Vβ T cells families indeed respond to tumor-specific antigens expressed on MOPC315.BM cells. Similar to human MM cells, MOPC315.BM cells express and secrete an idiotypic (Id) antibody and peptides from this antibody presented in association with MHC-Class I molecules would likely be one target recognized by the donor MT cells [29]. The induction of anti-MM-Id peptide responses has been studied following the vaccination of MM patients with autologous Id-pulsed dendritic cells [30] and a recent trial (#NCT01426828) aims to evaluate whether infusion of Id-KLH primed CD3/CD28 activated autologous lymphocytes mediates a clinically relevant Id-specific immunity.. Unfortunately, there is no information on other potential MOPC315.BM tumor specific molecules that might be recognized by MT cells. A search in several immunoinformatic databases (IMTG, VDJdb, McPAS-TCR) did not clearly indicate which MHC presented peptides might be bound by TCR bearing Vβ CDR3 2, 3 and 8.3 sequences. With regards to human MM there is currently no information on the myeloma-specific TCR sequence repertoire in MM patients [31].

The relative contribution of each Vβ family to the overall GvM response we observed is a subject for ongoing studies. Not all families may contribute equally to the GvM effect, possibly because only some of them are presented with dominant MHC-bound peptides [32, 33], or because they secrete cytokines that induce more effective anti-tumor responses. In another study, the Vβ13 family by itself was shown to dominate the B10.BR CD8 T-cell response against a myeloid leukemia cell line. Transplantation of these cell induced a slight GvT response with no concomitant acute GvHD [27].

Appropriate T-cell co-stimulation is critical for induction of effective anti-tumor T-cell function [24, 34–37]. Porter et al. [35] and Biavati et al. [38] showed that ex vivo co-stimulation of T cells via their CD3 and CD28 receptors can produce activated T cells that enhance the antitumor effect of donor lymphocyte infusions after allogeneic hematopoietic stem cell transplantation in patients with chronic myelogenous leukemia and MM. Noonan et al. were the first to report that infusion of autologous, ex vivo activated, marrow-infiltrating T cells could induce anti-tumor reactivity and enhance progression-free survival in MM patients, although there was no difference in overall survival [39]. Our results are in line with these findings. We saw that although in vitro activation of auto-MT cells led to target cell killing (Additional file 1: Figure S3) and transplantation of IL2/Ab stimulated auto-MT cells more than doubled the mean survival time (from 20 to 43 days, *p* < 0.0001), the mice eventually relapsed. The short-lived response after auto-MT infusion may be due to T cell exhaustion, a topic currently under intensive study [40, 41]. While appropriately activated (IL-2/Ab) allogeneic MT cells responded aggressively to target cells in vitro and induced long-term survival in vivo, on the other hand, transplantation of one dose of naïve allo-MT cells lead to only short-term clinical efficacy. Interruption of the GvM effect may have been due to development of effector T cell exhaustion because an additional infusion of naïve allo-MT cells was more effective allowing for long-term disease-free survival (Additional file 1: Figure S4, Fig. 4).

Another explanation for the lack of efficacy of auto-MT cells infusion may be the specificity of the Balb/c MT cells themselves. Flow cytometry clearly showed that co-culture with target cells resulted in overall expansion and activation of both CD4^+^ and CD8^+^ B10.D2 populations but only the CD4+ Balb/c population. While Balb/c T cells expressing 2, 3 and 8.3 Vβ family containing TCRs did become activated (but did not expand) they may not be the best anti-MOPC315.BM effector T cell clones and may only induce a weaker and short-lived GvM response. Transcriptome analysis of the Balb/c CD8^+^ T cell TCRs may reveal that other subfamilies are more effective. This may also be true in patients, however there is currently no data available to adequately address this question. A third explanation may be that the effectiveness of the allo- over auto-MT cell activity in our model is due to a miHA antigen (or antigens) recognized on the MOPC315.BM by B10.D2 but not Balb/c T cells (MOPC315 cells are Balb/c derived). These antigens would need to be different from the shared myeloma and allo-antigens reported by Binsfeld et al. that are recognized by TCRVβ families other than those used in our study [19]. A number of human leukemic restricted miHAs have been identified, including some on MM cells [42]. Some of these are capable of eliciting anti-tumor T cell responses [43] and indeed recent studies report development of engineered T cells bearing human miHA specific TCRs [44, 45]. Their activity towards MM has not been demonstrated.

## Conclusion

We have shown for the first time invocation of a strong and life-saving GvM response and prevention of GvHD by integrating auto-BMT with a ATCT composed only of transcriptome-identified MM reactive Vβ T cell families. With the use of new TCR sequencing technologies [46–48] it should become feasible to characterize, isolate and infuse tumor-specific donor T cell Vβ families into patients. This strategy is significant for MM therapy because it highlights the opportunity to develop a more effective treatment protocol combining a vigorous GvM response that eliminates residual MM cells in patients who have undergone pre-conditioning and auto-HSCT without inducing GvHD.

## Supplementary information

**Additional file 1 Figure S1**. Survival curve of Balb/c mice injected i.v. with MOPC315.BM (1x106, *n*= 4/group). **Figure S2.** *In vitro* reactivity of T-cells after 4-days co-culture with MOPC315.BM cells. **Figure S3**. Target cell cytotoxicity of activated B10.D2 or Balb/c vβ 2, 3 8.3 T cells. **Figure S4.** Monitoring of post-transplant reconstitution of spleen **(A)** and BM **(B)** T -cell subsets in normal Balb/c mice (*n*= 10/group) who received 6.5Gy irradiation and then autologous bone marrow transplantation (Auto-BMT). **Video S1.** Video of representative Balb/c mouse with hind leg paraplegia 35 days after i.v. injection with MOPC315.BM myeloma cells.

## Data Availability

The datasets supporting the conclusions of this article are included within the article and its additional file. For further information on original data, contact the Corresponding Author.

## Abbreviations

ASCT: Autologous stem cell transplantation
ATCT: Adoptive Allogeneic T-cell therapy
BMT: Bone marrow transplantation
CFSE: Carboxyfluorescein succinimidyl ester
GvHD: Graft versus host disease
GvM: Graft-versus-myeloma
MM: Multiple Myeloma
MST: Median survival times
MT cells: Myeloma-specific T cells
TCR: T cell receptor
