# Supplementary material for: Long-term survival without graft-versus-host-disease following infusion of allogeneic myeloma-specific Vβ T cell families
Source: J Immunother Cancer. 2019 Nov 14;7:301. doi: 10.1186/s40425-019-0776-9 (PMC6854718; doi:10.1186/s40425-019-0776-9)
Supplement: Supplementary file 1 — Additional file 1 Figure S1. Survival curve of Balb/c mice injected i.v. with MOPC315.BM (1x10^6^, n= 4/group). Figure S2. In vitro reactivity of T-cells after 4-days co-culture with MOPC315.BM cells. Figure S3. Target cell cytotoxicity of activated B10.D2 or Balb/c vβ 2, 3 8.3 T cells. Figure S4. Monitoring of post-transplant reconstitution of spleen (A) and BM (B) T -cell subsets in normal Balb/c mice (n= 10/group) who received 6.5Gy irradiation and then autologous bone marrow transplantation (Auto-BMT). Video S1. Video of representative Balb/c mouse with hind leg paraplegia 35 days after i.v. injection with MOPC315.BM myeloma cells. [file 40425_2019_776_MOESM1_ESM.zip › Supplementary Information JIC 1.pdf]

Infusion of allogeneic myeloma-specific Vβ T cell families leads to long-term survival without graft-versus-host-disease

Yado, S. [1], Luboshits, G.[1,3], Hazan, O.[2], Or, R[2] and Firer, MA[1.3.4#].

Supplementary data:

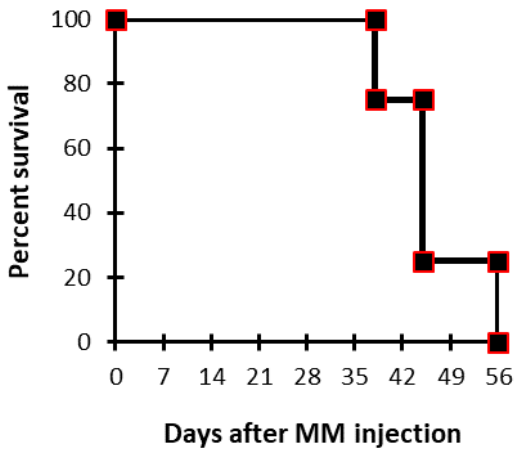

Supplementary Figure 1. Survival curve of Balb/c mice injected i.v. with MOPC315.BM ($1x10^6$, n= 4/group). All mice developed paraplegia at the endpoint due to spinal cord compression. For all injected mice, MOPC315.BM cells, identified as $CD4^+CD138^+$ positive cells, were detected in BM flushed from femurs and in the spleen. The experiment was repeated twice with three to five mice per group. and one representative result is shown.

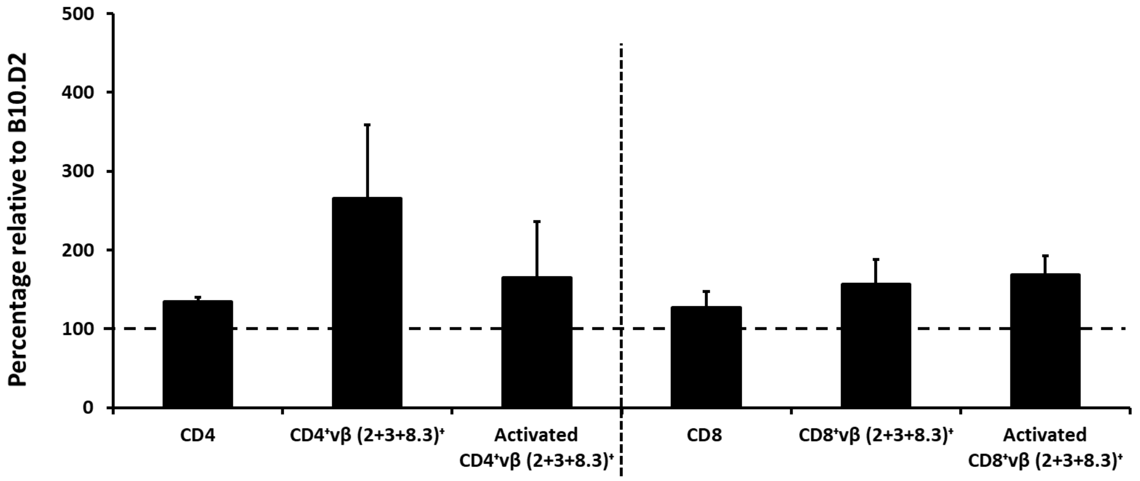

Supplementary Figure 2. *In vitro* reactivity of T-cells after 4-days co-culture with MOPC315.BM cells. Relative percentages (mean ± SD) i.e. actual percentages normalized to those of non-activated B10.D2 spleenocytes alone, are represented for 2 independent experiments of 4-day co-cultures of B10.D2 spleenocytes alone or together with Mitomycin-C-treated MOPC315.BM cells). Activated cells displayed a $CD69^{+}$ phenotype. On the left, percentages of CD4 T cells (within $CD3^{+}$), $CD4^{+}$vβ $(2+3+8.3)^{+}$ double positive T cells (within $CD3^{+}$) and activated $CD4^{+}$vβ $(2+3+8.3)^{+}$ T cells ($CD69^{+}$ within $CD4^{+}$vβ $(2+3+8.3)^{+}$) and, on the right, percentages of CD8 T cells (within $CD3^{+}$), $CD8^{+}$vβ $(2+3+8.3)^{+}$ double positive T cells (within $CD3^{+}$) and activated $CD8^{+}$vβ $(2+3+8.3)^{+}$ T cells ($CD69^{+}$ within $CD8^{+}$vβ $(2+3+8.3)^{+}$).

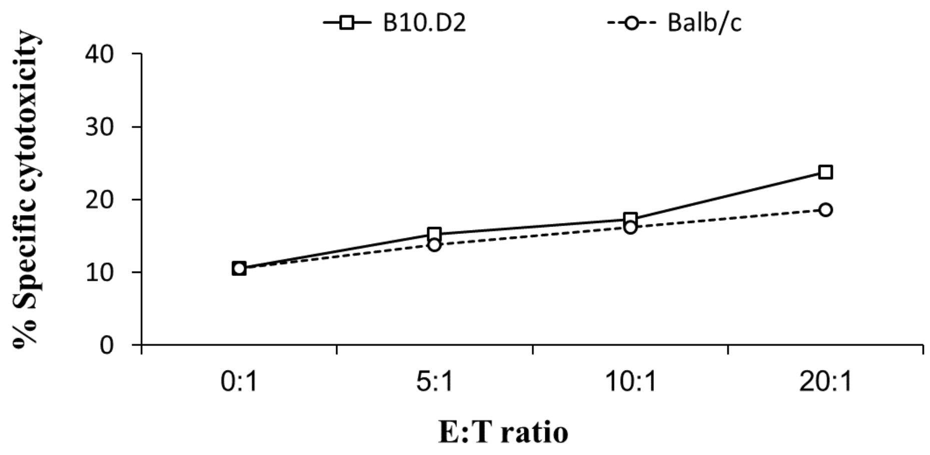

Supplementary Figure 3. Target cell cytotoxicity of activated B10.D2 or Balb/c vβ 2, 3 8.3 T cells. MOPC315.BM cells (Target cells (T)) were labeled with CFSE and co-cultured with B10.D2 or Balb/c vβ 2, 3, 8.3 positive T cells (Effector cells (E)) for 4 hours at the ratios indicated. At the end of the experiment, dead cells were labeled with Sytox blue and the percentage of MOPC315.BM cell death was analyzed by flow cytometry. Values shown as mean ± SD of triplicate cultures. One representative of 2 independent experiments is shown.

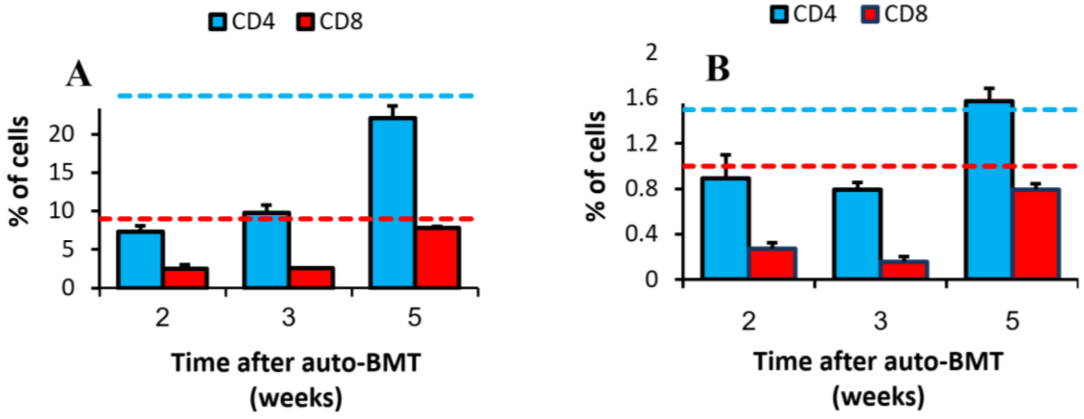

Supplementary Figure 4. Monitoring of post-transplant reconstitution of spleen **(A)** and BM **(B)** T -cell subsets in normal Balb/c mice (n= 10/group) who received 6.5Gy irradiation and then autologous bone marrow transplantation (Auto-BMT). Dashed lines: % of CD4 (**Blue**) and CD8 (**Red**) cells in healthy Balb/c mice. Two, 3 and 5 weeks after auto-BMT, BM and spleens from representative animals were collected and analyzed by flow cytometry. Following conditioning therapy and auto-BMT, mice underwent an "aplastic phase" until the immune system was reconstituted following auto-BMT. By 5 weeks after auto-BMT, the percent of CD4 and CD8 T-cell subpopulations in both spleen and BM had reached normal levels. All remaining mice survived until termination of the experiment (42 days after auto-BMT).

Paraplegic mice.wmv

Video 1

Video 1. Video of representative Balb/c mouse with hind leg paraplegia 35 days after i.v. injection with MOPC315.BM myeloma cells.
